# Longitudinal association of oral functions and dementia in Japanese older adults

**DOI:** 10.1038/s41598-024-56628-8

**Published:** 2024-03-11

**Authors:** Komei Iwai, Tetsuji Azuma, Takatoshi Yonenaga, Yasuyuki Sasai, Taketsugu Nomura, Iwane Sugiura, Yujo Inagawa, Yusuke Matsumoto, Seiji Nakashima, Yoshikazu Abe, Takaaki Tomofuji

**Affiliations:** 1 Department of Community Oral Health, School of Dentistry, Asahi University, 1-1851 Hozumi, Mizuho, Gifu, 501-0296 Japan; 2 Gifu Dental Association, 1-18 Minamidori, Kano-cho, Gifu, 500-8486 Japan

**Keywords:** Health care, Medical research, Risk factors

## Abstract

The relationship between oral functions and dementia was examined in 7384 older adults (age ≥ 75 years) who visited a dental clinic in Gifu, Japan. Participants without dementia in a baseline survey in April 2018 were followed until March 2021. As oral functions, chewing function, tongue and lip function, and swallowing function were assessed by self-administered questionnaire, by oral diadochokinesis test, and by repetitive saliva swallowing test, respectively. The presence of systemic diseases was based on data obtained from the National Database of Health Insurance of Japan. At follow-up, 415 (6%) participants were diagnosed with dementia. Multivariate logistic regression analyses showed the presence of dementia at follow-up was associated with female (odds ratio [OR] 1.386; 95% confidence interval [CI] 1.117–1.719), age (OR 1.078; CI 1.056–1.101), regular dental checkups (absence; OR 1.452; CI 1.180–1.788), brushing frequency ≥ twice/day (absence; OR 1.510; CI 1.194–1.911), decayed teeth (presence; OR 1.328; CI 1.071–1.648), swallowing function (poor; OR 1.484; CI 1.135–1.939) at baseline. It was found that poor swallowing function was associated with the future onset of dementia.

## Introduction

In Japan, the population is aging more rapidly than in other countries^1^, and as of 2021, there were approximately 18.7 million older adults aged ≥ 75 years, accounting for nearly 15% of the total population^2^. The important issue in an aging society is the increasing prevalence of physical and mental disabilities with age^3,4^. Of the physical and mental disabilities with age, the proportion of older adults with dementia has been increasing in recent years, with a report in 2020 showing that 20% of older adults aged ≥ 75 years had dementia, which has become one cause of pressure on social security costs in Japan^5^. Dementia is a disease in which once normally developing intelligence is irreversibly decreased due to organic brain damage^6,7^. Dementia is often confused with age-related memory impairment^8^. However, it becomes more pronounced memory impairment, disorientation, and cognitive dysfunction as symptoms progress^9,10^. In addition, no fundamental treatment for dementia has yet been developed^11^. Therefore, it is very important to investigate factors related to dementia and explore immediate countermeasures.

Studies have reported the relationship between oral status and dementia. For example, it has been reported that tooth loss and occlusion affect brain function and trigger the onset of dementia^12^. It is also known that alterations of the oral microbiota causing chronic periodontal disease are associated with the risk of dementia^13^. In a longitudinal study, it was reported that poor periodontal health and tooth loss appear to increase the risk of both cognitive decline and dementia^14^. However, these reports demonstrated associations between specific oral diseases and dementia, and it is not clear from these diseases which oral functions are impaired and thus bring on dementia. Therefore, further studies investigating the longitudinal relationships between oral functions and dementia are needed.

In Japan, the National Database of Health Insurance of Japan (NDB) provides a database of the state of people with dementia. In addition, dental checkups including oral functions are conducted once a year for older adults aged ≥ 75 years in Gifu, Japan, and the results of oral functions are also compiled in a database. Therefore, by combining these data, it is possible to analyze the relationship between oral functions and dementia. Oral functions include chewing, swallowing, and tongue function, and the relationship between oral functions and dementia may differ according to the types of oral functions. Therefore, this was a longitudinal study over a period of two years in which the aim was to clarify the longitudinal relationships between three types of oral functions and dementia in Japanese older adults aged ≥ 75 years.

## Methods

### Participants

**Figure 1:**
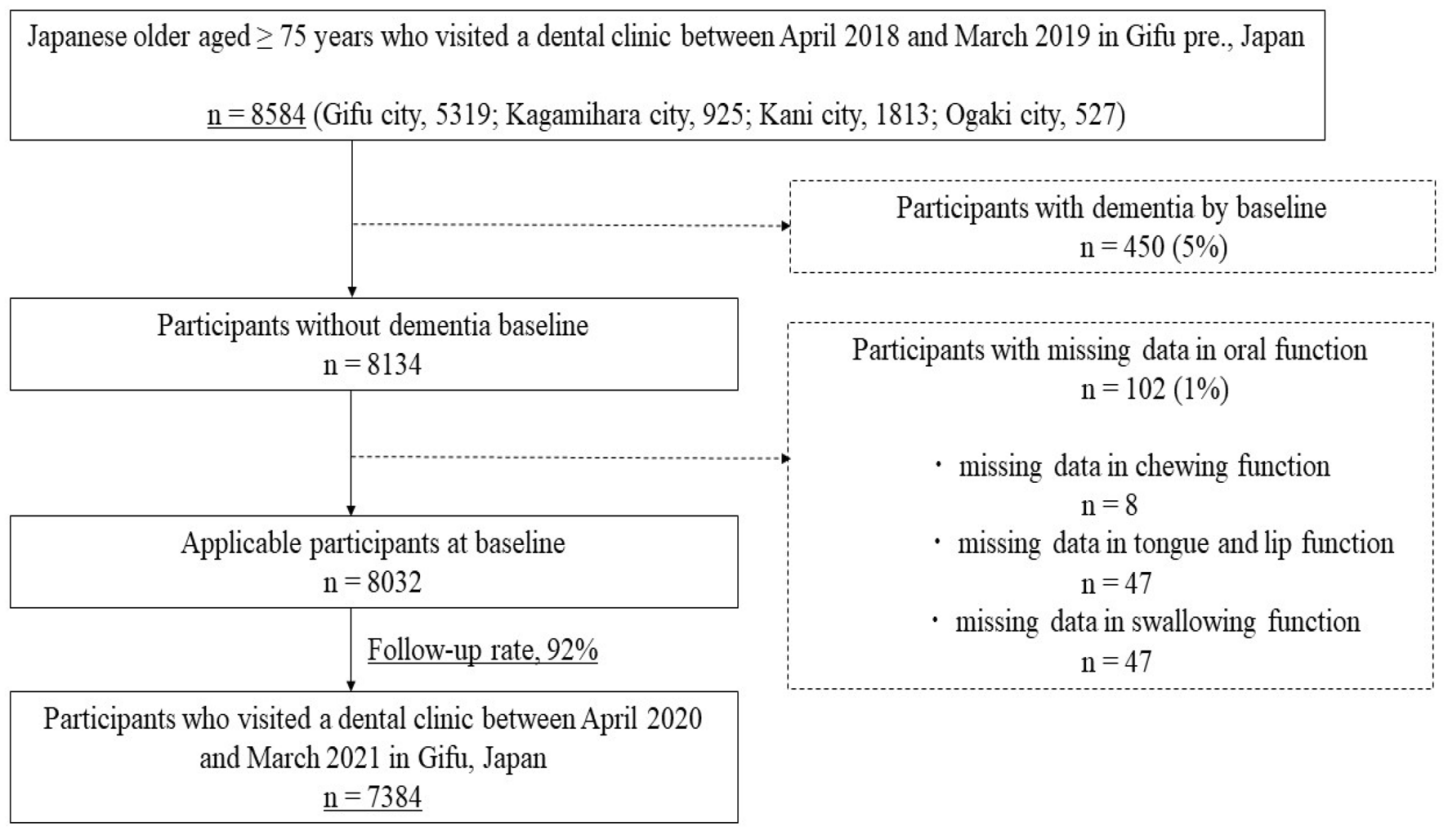
Flowchart of data selection criteria.

Data from community residents who received dental checkups in Gifu City, Kagamihara City, Kani City, and Ogaki City in Gifu, Japan, were analyzed. Between April 2018 and March 2019, a total of 8584 Japanese older adults aged ≥ 75 years participated in the baseline survey. First, participants with dementia at baseline (450 participants) were excluded. Furthermore, participants with missing data for oral functions, including participants with missing data for chewing function (8 participants), tongue and lip function (47 participants), and swallowing function (47 participants), were excluded from the analysis. Of these 8032 participants, 7384 were followed from April 2020 to March 2021 (follow-up rate, 92%). Therefore, the data of 7384 community residents (3078 men and 4306 women, mean age 80.0 years) were analyzed in the present study (Fig. 1).

### Survey items in the National Health Insurance database system

The information about sex, age, and presence or absence of hypertension, diabetes mellitus, and dementia was obtained from the National Database of Health Insurance of Japan (NDB)^15^. Presence or absence with dementia was investigated both at baseline and follow-up, and Participants with dementia at follow-up selected as outcome.

### Smoking habits and oral items

Data on the following smoking habits and oral items were obtained at baseline: presence or absence of regular dental checkups, presence or absence of brushing frequency ≥ twice/day, presence or absence of number of present teeth ≥ 20, presence or absence of decayed teeth, presence or absence of periodontal pockets ≥ 4 mm, chewing function, tongue and lip function, and swallowing function. The data for the oral items were provided by the Gifu Dental Association, Japan. For smoking habits, participants who smoked at least one cigarette per day were included (presence or absence)^15,16^. Regular dental checkups were considered to involve regular visits to the dental clinic (at least once every 6 months or less than once every 6 months)^15,17^. The number of decayed teeth was calculated as D (Decayed), missing teeth as M (Missing), and filled teeth as F (Filled), and the number of DMF teeth was used to evaluate dental caries history^15,18^. The coded values of the Community Periodontal Index (CPI) were used to evaluate periodontal pockets ≥ 4 mm, with codes 1 and 2 being evaluated as periodontal pockets ≥ 4 mm^15,19^. Chewing function was assessed by difficulty eating hard food. Participants were asked to choose from “It is more difficult to eat hard food than it was six months ago (presence or absence)” in the self-administered questionnaire^15,20^. Tongue and lip function was assessed using the oral diadochokinesis test for tongue and lip function, with poor tongue and lip function defined as less than 30 syllables in 5 s of any one of “Pa”, “Ta”, or “Ka”^15,21^. For swallowing function, those who swallowed less than 3 times in 30 s in the repetitive saliva swallowing test were evaluated as having poor swallowing function^15,22^.

### Statistical analysis

The Kolmogorov–Smirnov test was used to check the normality of the data. Because all continuous variables were not normally distributed, data are expressed as medians (first and third quartiles). The chi-squared test was used to evaluate significant differences in characteristics of oral functions with and without dementia. Univariate and multivariate logistic regression analyses were performed with presence of dementia as the dependent variable. In the multivariate stepwise logistic regression analysis, variables with *p* > 0.10 were excluded from the model^23^. Additionally, variables that were significantly different in univariate logistic regression analysis were selected into a third category in addition to gender and age. A Hosmer–Lemeshow fit test was performed to confirm the goodness of fit of our model. A statistical analysis software (SPSS statistics version 27; IBM Japan, Tokyo, Japan) was used to analyze all data. A *p* values < 0.05 were considered significant^15^.

### Research ethics

**Figure 2:**
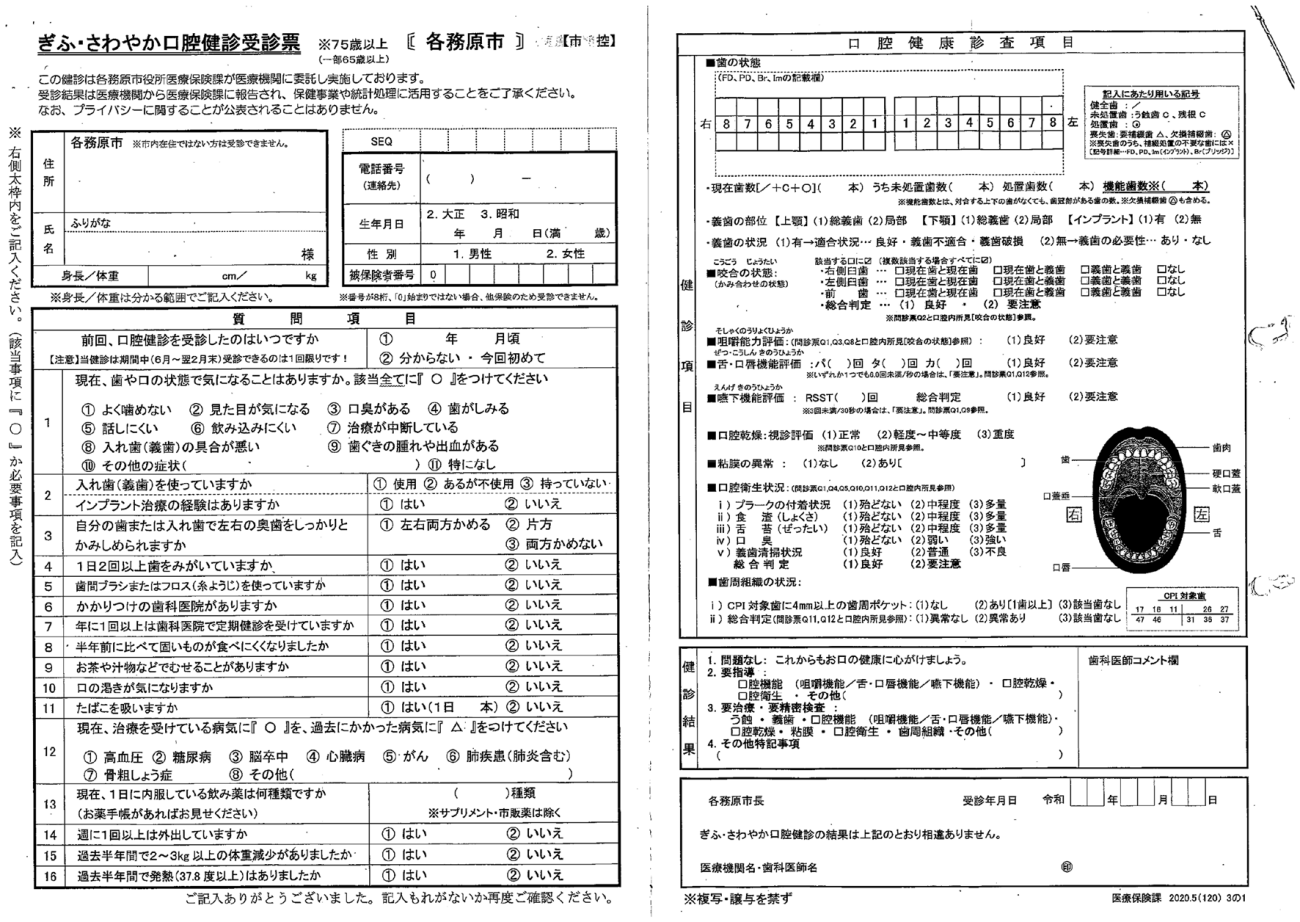
The confirmation document form used to obtain informed consent.

Our study is an experiment involving human participants and informed consent has been obtained in writing from all participants and/or their legal guardians, and the confirmation document is attached separately (Fig. 2). Our study was approved by the Ethics Committee of Asahi University (No. 33006) and was performed in accordance with the Declaration of Helsinki (as revised in Brazil 2013).

## Results

Table 1 shows the characteristics of the participants at baseline and at follow-up. Overall, there were 3078 males (42%) and 4306 females (58%). The proportion of participants with hypertension (*p* < 0.001) was significantly higher at follow-up than at baseline. In addition, the proportion of participants with number of present teeth ≥ 20 (*p* < 0.001) was significantly lower at follow-up than at baseline. However, there were no significant differences in the other factors, except the proportions of those with hypertension and with number of present teeth ≥ 20, between at baseline and at follow-up.

**Table 1:** Participants’ characteristics (n = 7384).

Factor	Baseline	Follow-up	*p* value*
Gender^a^	4306 (58%)	4306 (58%)	–
Age (years)	80 (77, 84)	82 (79, 86)	–
Smoking habits^b^	128 (2%)	125 (2%)	0.849
Hypertension^b^	4237 (57%)	4759 (65%)	< 0.001
Diabetes^b^	2553 (35%)	2632 (36%)	0.173
Regular dental checkups^b^	5167 (70%)	5149 (70%)	0.747
Brushing frequency (times/day)			
-1	1407 (19%)	1426 (19%)	0.691
2-	5977 (81%)	5958 (81%)	
Number of present teeth (tooth)			
-19	2518 (34%)	2727 (37%)	< 0.001
20-	4866 (66%)	4657 (63%)	
Decayed teeth^b^	1961 (27%)	1873 (25%)	0.099
Periodontal pockets (mm)			
-3	2432 (33%)	2360 (32%)	0.206
4-	4952 (67%)	5024 (65%)	
Chewing function^c^	1816 (25%)	1873 (25%)	0.279
Tongue and lip function^c^	2310 (31%)	2397 (33%)	0.124
Swallowing function^c^	974 (13%)	1016 (14%)	0.311

^a^Female (proportion of female);

^b^Presence (proportion of presence);

^c^Poor (proportion of poor).

*p < 0.05, using Fishers exact test.

Table 2 shows the characteristics of oral functions at baseline by with and without dementia at follow-up. In the present study, 415 participants (6%) were newly diagnosed with dementia at follow-up. Participants with dementia were characterized by a significantly higher proportion of poor tongue and lip function (*p* = 0.028) and poor swallowing function (*p* < 0.001) than those without dementia. On the other hand, there was no significant association between chewing function and dementia.

**Table 2:** Baseline characteristics regarding oral functions of the study participants with and without dementia at follow-up.

Factor	Dementia		*p* value*
	Absence n = 6969	Presence n = 415	
Chewing function			
Well	5266 (75%)	302 (73%)	0.202
Poor	1703 (25%)	113 (27%)	
Tongue and lip function			
Well	4809 (69%)	265 (64%)	0.028
Poor	2160 (31%)	150 (36%)	
Swallowing function			
Well	6082 (87%)	328 (79%)	< 0.001
Poor	887 (13%)	87 (21%)	

*p < 0.05, using Fishers exact test.

Table 3 shows crude odds ratio (OR) and 95% confidence interval (CI) for dementia at follow-up. The results showed that the risk of dementia after two years was significantly correlated with female (OR 1.368; 95% CI 1.112 to 1.684), age (OR 1.096; 95% CI 1.075 to 1.117), hypertension (presence; OR 1.304; 95% CI 1.062 to 1.601), regular dental checkups (absence; OR 1.620; 95% CI 1.322 to 1.984), brushing frequency ≥ twice/day (absence; OR 1.601; CI 1.277–2.006), number of present teeth ≥ 20 (absence; OR 1.337; CI 1.092–1.636), decayed teeth (presence; OR 1.490; CI 1.208–1.837), tongue and lip function (poor; OR 1.206; CI 1.025–1.549), and swallowing function (poor: OR 1.819; 95% CI 1.421 to 2.327) at baseline. On the other hand, there was no significant association between dementia after two years and chewing function at baseline.

**Table 3:** Crude OR and 95% CI for dementia at follow-up.

Factor	OR	95% CI	*p* value
Gender			
Male	1	(Reference)	0.003
Female	1.368	1.112–1.684	
Age (years)	1.096	1.075–1.117	< 0.001
Smoking habits			
Absence	1	(Reference)	0.399
Presence	0.679	0.276–1.669	
Hypertension			
Absence	1	(Reference)	0.011
Presence	1.304	1.062–1.601	
Diabetes			
Absence	1	(reference)	0.100
Presence	1.187	0.968–1.456	
Regular dental checkups			
Presence	1	(Reference)	< 0.001
Absence	1.620	1.322–1.984	
Brushing frequency ≥ twice/day			
Presence	1	(Reference)	< 0.001
Absence	1.601	1.277–2.006	
Number of present teeth ≥ 20			
Presence	1	(Reference)	0.005
Absence	1.337	1.092–1.636	
Decayed teeth			
Absence	1	(Reference)	< 0.001
Presence	1.490	1.208–1.837	
Periodontal pockets ≥ 4 mm			
Absence	1	(Reference)	0.497
Presence	1.075	0.873–1.324	
Chewing function			
Well	1	(Reference)	0.202
Poor	1.156	0.925–1.445	
Tongue and lip function			
Well	1	(Reference)	0.028
Poor	1.206	1.025–1.549	
Swallowing function			
Well	1	(Reference)	< 0.001
Poor	1.819	1.421–2.327	

*OR* odds ratio, *CI* confidence interval.

Table 4 shows adjusted OR and 95% CI for dementia at follow-up. After adjusting for after adjusted for gender, age, hypertension, regular dental checkups, brushing frequency ≥ twice/day, number of present teeth ≥ 20, decayed teeth, tongue and lip function, and swallowing function, the risk of dementia at two years was significantly correlated with female (OR 1.386; 95% CI 1.117–1.719), age (OR 1.078; CI 1.056–1.101), regular dental checkups (absence; OR 1.452; CI 1.180–1.788), brushing frequency ≥ twice/day (absence; OR 1.510; CI 1.194–1.911), decayed teeth (absence; OR 1.328; CI 1.071–1.648), swallowing function (poor; OR 1.484; CI 1.135–1.939) at baseline. However, chewing function and tongue and lip function were not associated with the presence of dementia.

**Table 4:** Adjusted OR and 95% CI for dementia at follow-up.

Factor	OR	95% CI	*p* value
Gender			
Male	1	(Reference)	0.003
Female	1.386	1.117–1.719	
Age	1.078	1.056–1.101	< 0.001
Hypertension			
Absence	1	(Reference)	0.554
Presence	1.067	0.862–1.320	
Regular dental checkups			
Presence	1	(Reference)	< 0.001
Absence	1.452	1.180–1.788	
Brushing frequency ≥ twice/day			
Presence	1	(Reference)	< 0.001
Absence	1.510	1.194–1.911	
Number of present teeth ≥ 20			
Presence	1	(Reference)	0.702
Absence	1.042	0.843–1.289	
Decayed teeth			
Absence	1	(Reference)	0.010
Presence	1.328	1.071–1.648	
Tongue and lip function			
Well	1	(reference)	0.771
Poor	0.967	0.773–1.211	
Swallowing function			
Well	1	(Reference)	0.004
Poor	1.484	1.135–1.939	

Adjustment for gender, age, hypertension, regular dental checkups, brushing frequency ≥ twice/day, number of present teeth ≥ 20, decayed teeth, tongue and lip function, and swallowing function.

*OR* odds ratio, *CI* confidence interval.

## Discussion

To the best of our knowledge, this is the first longitudinal study to examine the associations between swallowing function and dementia in Japanese older adults using data from the NDB. The results showed that participants with dementia after two years of follow-up had a higher proportion of poor swallowing function at baseline than those without dementia. The results of logistic regression analysis showed that, after adjusting for gender, age, hypertension, regular dental checkups, brushing frequency ≥ twice/day, number of present teeth ≥ 20, decayed teeth, and tongue and lip function, the presence or absence of dementia after 2 years was associated with swallowing function at baseline. From these results, it was predicted that a decrease in swallowing function was associated with a higher risk of the onset of dementia in the future.

There are several possible mechanisms for the relationship between swallowing function and dementia. Swallowing actions activate brain function and improve learning and memory skills^24,25^. It was also reported that swallowing actions increase cerebral blood flow and the partial pressure of oxygen in the brain^26^. Decreased cerebral blood flow is a known risk factor for dementia^27^. Thus, participants with swallowing difficulties might have a greater risk for dementia because of reduced cerebral blood flow and even ischemia. In addition, people with poor swallowing function tend to consume less fruit and vegetables and more high-energy foods than those with good swallowing function^28^. High-calorie diets rich in carbohydrates and saturated fatty acids tend to increase the risk of dementia^29^. Therefore, these may be also among the mechanisms. However, further research is needed to clarify the mechanisms by which poor swallowing function is associated with dementia.

In the present study, chewing function was not associated with dementia. A previous report showed an association between chewing function using the evaluation of chewing function was based on muscle activity of the masticatory muscles and dementia^30^. The results in the previous and present studies may have been different due to their different measurement methods. In the future, we would like to consider judging chewing function by methods such as assessing the distribution of crushed particles in chewing samples (e.g., gummy jellies), the amount of elution of contents in chewing samples, or the activity of the masticatory muscles.

In addition, a cross-sectional study reported an association between poor tongue and lip function and dementia using the same research methodology as the present study^31^. This finding differs from the present study. This may be related to differences in research methods (cross-sectional vs. longitudinal studies). It may also be related to the bias in the proportion of participants with poor tongue and lip function at baseline. In the previous study, most participants possessed good tongue and lip function^31^, unlike the present study. Therefore, external validity should be considered in this study.

In the present study, lack of regular dental checkups and decayed teeth were associated with dementia. Past reports showed that recommendations for regular dental checkups reduced the risk of dementia onset^32^. Furthermore, it was reported that mutans bacteria that cause tooth decay adhere to the walls of blood vessels in the brain and reduce cognitive function^33^. These previous studies support the results of the present study. These studies reported that maintaining a good oral environment and controlling oral bacteria decrease the risk of dementia onset. In the present study, brushing frequency ≥ twice/day was associated with dementia. It is widely known that proper brushing habits are important for preventing decayed teeth and maintaining a good oral environment^34,35^. Therefore, participants in the present study who brushed ≥ twice/day may have engaged in proper brushing habits, had a good oral environment, and may not have had decayed teeth, and thus had a lower risk of dementia onset. Therefore, brushing habits may indirectly contribute to delaying the onset of dementia via maintaining a good oral environment.

There was no association between number of present teeth and dementia in our study. Past studies were reported that missing tooth is associated with development of Alzheimer's dementia^36^. This may be related to number of participants with missing tooth. In our study, proportion of participants with number of present teeth ≥ 20 was 66%, which is very higher than average in older adults aged ≥ 75 years in Japan (51.6%)^37^. Therefore, it is possible that there was no association between number of present teeth and dementia because number of participants with missing tooth was too small.

A major strength of the present study is its sample size of more than 7000 Japanese older adults. In addition, it was a longitudinal study, which is useful for establishing a causal relationship between dementia and poor swallowing function and for inferring factors that contribute to pressure on social security costs in Japan. Furthermore, it was possible to gather study population data from multiple locations in Gifu, Japan (Gifu City, Kagamihara City, Kani City, and Ogaki City).

In the present study, the Hosmer–Lemeshow fit test was used in a multivariate logistic regression analysis model. The Hosmer–Lemeshow fit test is used to examine the fit of a multivariate logistic regression analysis model and tests whether the observed event rate in a subgroup model fits the expected event rate. The Hosmer–Lemeshow test is considered to show a good fit with *p* values > 0.05^38^. In the present study, the *p* value was 0.288, suggesting a good fit.

However, there are several limitations to the present study. First, since participants of the present study visited to dental checkups, they may have been a highly health-conscious population. In our study, 8.6% of all participants were certified as needing supports and 6.9% were certified as needing cares based on Long-Term Care Insurance Law in Japan at baseline. This was a low value compared to proportion with certified as needed supports (8.8%) and needed cares (23.1%) in older adults aged ≥ 75 years in Japan^2^. Second, the presence or absence of diseases not in the database is unknown, since the NDB was used. Finally, our study is a longitudinal study, but may be the potential reversal of cause and effect. For example, certain types of dementia, like Parkinson’s-associated dementia and vascular dementia, are known to affect swallowing function, even in the preclinical stage of cognitive dysfunction.

In conclusion, the present study showed that Japanese older adults with poor swallowing function have a higher risk for future dementia.

## Data Availability

The data that support the findings of this study are available from “Gifu National Health Insurance Federation” and “Wide-Area Federation of Medical Care for Late-Stage Older People” but restrictions apply to the availability of these data, which were used under license for the current study, and so are not publicly available. Data are however available from the authors upon reasonable request and with permission of “Gifu National Health Insurance Federation” and “Wide-Area Federation of Medical Care for Late-Stage Older People”.
